# Female-controlled dual protection methods: Prevalence, predictors, experiences and perceptions among young women living with HIV in northern Uganda–A mixed-method study protocol

**DOI:** 10.1371/journal.pone.0290338

**Published:** 2023-10-09

**Authors:** Edward Kumakech, Joy Acen, Marvin Musinguzi, Doryn Ebong, James Okello

**Affiliations:** 1 Department of Nursing, Faculty of Nursing and Midwifery, Lira University, Lira City, Uganda; 2 Department of Midwifery, Faculty of Nursing and Midwifery, Lira University, Lira City, Uganda; 3 Department of Community Health, Faculty of Public Health, Lira University, Lira City, Uganda; 4 Department of Nursing and Midwifery, Lira Regional Referral Hospital, Lira City, Uganda; 5 Department of Obstetrics and Gynecology, Lira Regional Referral Hospital, Lira City, Uganda; The AIDS Support Organization, UGANDA

## Abstract

**Study background:**

The use of dual protection methods among young women living with HIV (YWLHIV) aged 15–24 years in sub-Saharan Africa (SSA) is poorly researched despite the double risk of unintended pregnancy and HIV. Even more scanty is literature on the use of female-controlled dual protection methods. We propose to determine the female-controlled dual protection prevalence, and the predictors among YWLHIV in northern Uganda. The study will also explore the YWLHIV’s experiences and perceptions regarding the female-controlled dual protection methods.

**Materials and methods:**

This study will employ a mixed-methods design. The study area will be Lira district and Lira city located in northern Uganda. The setting for recruitment of participants will be the public health facility–based anti-retroviral therapy (ART) clinics. These ART clinics serves a total of about 1,771 YWLHIV. A sample of 425 YWLHIV will be selected by stratified random sampling from the ART clinic registers. The three strata of interest will be the YWLHIV attending the ART clinic at referral hospitals, health centers level IV (primary healthcare centers), and health centers level III (dispensaries). The primary outcome will be the use of the female-controlled dual protection methods. The outcome will be measured by asking the YWLHIV ‘what methods under their control as YWLHIV do they use to protect against both unintended pregnancy and HIV during sexual intercourse with their male partners.’ The questionnaire also has measures for the unintended pregnancy, HIV status, and the potential predictors. Qualitative component of the study will be in-depth interviews of the participants about their experiences and perceptions regarding the female-controlled dual protection methods. Data collection was still ongoing at the time of first submission of this study protocol to the journal (14th March 2023). The Statistical Package for Social Sciences (SPSS) version 23.0 will be used for the statistical analyses. Descriptive statistics, bivariate and multivariate regression analyses will be used to establish the prevalence, associated factors and the predictors of the outcome respectively. The statistical significance level of 5% and 95% confidence interval will be considered. In-depth interviews will be manually analyzed using a thematic analysis approach for codes, themes, and categories.

## Introduction

HIV burden among young women in sub-Saharan Africa (SSA) is a significant concern. Despite representing only 10% of the population, adolescent girls and young women (AGYW) aged 15–24 accounted for 25% of HIV infections in 2020 [1]. In SSA, many sexually active AGYW living with HIV do not disclose their HIV status to their partners (76–100%) and fail to use protection (35–55%), primarily due to fear of stigma and discrimination [2, 3]. The risk of sexual transmission of HIV among discordant couples involving young women living with HIV (YWLHIV) is also high, with studies reporting transmission rates ranging from 0.0007 to 0.0082 per coital act [4, 5].

Unintended pregnancy is another significant challenge faced by young women in SSA. The prevalence of unintended pregnancy among women of reproductive age is as high as 44.2%, with YWLHIV more likely to report unwanted pregnancy compared to their counterparts [6–8]. Studies have found prevalence rates of unintended pregnancy ranging from 45% to 73.6% among women living with HIV [9–12].

Low contraceptive use, especially modern methods like condoms and hormonal contraceptives, contributes to the high prevalence of unintended pregnancy among YWLHIV. For example, in Uganda, only 39.8% of adolescent girls and young women use modern contraceptives, and male condom use is as low as 19.7% [13]. Similarly, in South Africa, only 12.4% of the adolescent mothers living with HIV were found to use male condoms [14].

To address the dual risks of HIV transmission and unintended pregnancy, various dual protection methods have been implemented. These methods include the use of male condoms, hormonal contraceptives, and antiretroviral drugs (ARVs) alone or in combination. Dual protection methods aim to prevent HIV/sexually transmitted infections (STIs) and unintended pregnancy simultaneously [14, 15]. However, the use of male condoms for dual protection [16] is limited, with low rates reported among AGYW and AGYWLHIV in countries like Uganda and Zimbabwe [17, 18]. Furthermore, relying solely on condoms for contraception is not as effective as other modern contraceptive options in preventing unintended pregnancy [19].

Female-controlled dual protection methods, such as female condoms and hormonal contraceptives combined with ARV drugs like pre-exposure prophylaxis (PrEP) or post-exposure prophylaxis (PEP), have been introduced. However, data on the use of these methods among AGYWLHIV in SSA is scarce. Studies in Zambia have highlighted the low use of dual protection methods among HIV discordant couples [20]. The use of hormonal contraceptive methods with PrEP or PEP by AGYWLHIV is supported by national policy guidelines in Uganda [21, 22]. These ARV drugs have shown efficacy in preventing HIV transmission, with Tenofovir disoproxil fumarate-Emtricitabine (TDF-FTC) as PrEP reducing the risk of HIV infection by 44% to 75% when used consistently [23–30].

Contraceptive methods like implants, intrauterine devices, and injectable have low failure rates and are recommended for dual protection [19]. However, traditional methods like withdrawal and periodic abstinence have higher failure rates. Young women living with HIV desire to bear children [14, 31] and yet face challenges in adhering to dual protection methods as per the recommendations of the World Health Organization [32], particularly when male condoms are demanded by their partners. Female-controlled dual protection methods including the use of ARV drugs like PrEP and PEP show promise but require further research.

### Research questions

1. What is the level of use of the female-controlled dual protection methods among the AGYWLHIV?
2. What are the predictors for the use of the female-controlled dual protection methods among the AGYWLHIV?
3. What are the experiences and perceptions regarding the use of the female-controlled dual protection methods among the AGYWLHIV?

### Specific objectives

1. To determine the female-controlled dual protection prevalence among the YWLHIV.
2. To identify the predictors for the use of female-controlled dual protection methods among the YWLHIV.
3. To explore the experiences and perceptions regarding the use of the female-controlled dual protection methods amongst the YWLHIV.

### Study significance

The study will generate data on the female-controlled dual protection prevalence, and predictors among the YWLHIV in a SSA country Uganda. The study will also generate data on the YWLHIV in Uganda’s experiences, perceptions and perspectives on the factors influencing the use of female controlled dual protection methods including the use of ARV drugs for the prevention of both HIV transmission and unintended pregnancy. These study findings will inform policies for the integrated delivery of safer conception, contraceptive methods and antiretroviral therapy services to YWLHIV in SSA. The study findings will also inform quality improvement efforts on existing interventions such as sexual contact tracing, testing and treatment for HIV, couple testing and treatment for HIV, couple counselling against HIV/AIDS stigma and discrimination, PrEP, PEP, and the sexual reproductive health of YWLHIV among others.

## Materials and methods

### Study design

This will be a mixed-methods study specifically an embedded mixed-method design. It is a design in which one data set provides a supportive secondary role in a study based primarily on the other data set [33]. The primary study will be the quantitative study on the female-controlled dual protection prevalence and the predictors for its use among the YWLHIV in Uganda. The qualitative study will generate the secondary data on perceptions of the rationales for the non-use of dual protection methods among the YWLHIV attending ART clinic in Uganda.

### Study area and setting

The study will be conducted in Lira district and Lira city. Lira district and Lira city are located in northern Uganda at a distance of about 340 kilometers from Kampala the capital city of Uganda. It is bordered by Alebtong district to the East, Kole district to the West, Apac district to the Southwest, Dokolo district to South East, Otuke district to the Northeast, and Pader district to the North. Lira district and Lira city will be the study area because of the high prevalence of HIV among AGYW in the area. The high rate of unprotected sexual intercourse among YWLHIV as evidenced by the high prevalence of unintended pregnancy among them standing at 42.7% [34] makes the study area suitable for the study of the female-controlled dual protection prevalence and its predictors. The HIV prevalence among the 15–49 years in Lira district and Lira city located in mid-northern Uganda stood at 7.2% by 2020 which was higher than the national average of 5.4% [35]. The setting within Lira district and Lira city where the participants will be recruited from will be the ART clinics of the six public health facilities in Lira district and Lira city namely Lira Regional Referral Hospital (LRRH), Lira University Hospital (LUH), Ober health center, Ogur health center, Amuca health center, and Barapwo health center. These ART clinics together serve 1,771 YWLHIV aged 15–24 years as of September 2022.

### Study population

The study population will be YWLHIV aged 15–24 years in Lira district and Lira city northern Uganda. The accessible population for study will be the YWLHIV aged 15–24 years who are attending the ART clinics at public health facilities in Lira district and Lira city northern Uganda. Lira district and Lira city has an estimated 28,000 adult persons living with HIV (PLHIV) of 15+ years of age [36] which was about 2% of the total 1,400,000 PLHIV in Uganda [35]. Only 82.5% and 80.0% of the PLHIV aged 15–64 years in mid-northern region of Uganda were receiving ART and were virally suppressed respectively which were lower than the national averages of 90% and 82% respectively [37].

### Inclusion criteria

The YWLHIV aged 15–24 years who have been living with HIV for 1+ years and report to be sexually active even if just one-off sexual intercourse in the past 1 year and are residents of Lira district or Lira city will be included in the study.

### Exclusion criteria

The sexually active YWLHIV aged 15–24 years who are residents of Lira district or Lira city will be excluded from the study if they are (a) without uterus such as from total abdominal hysterectomy and so cannot conceive unintended pregnancy; (b) sterile or infertile and so cannot conceive unintended pregnancy; (c) engaged to male sexual partners who have undergone male sterilization (vasectomy) and so cannot conceive unintended pregnancy; (d) deaf or mute and so cannot communicate in the interviews; (e) visibly too sick physically or mentally to withstand the study procedures.

### Sample size determination

The sample size will be 424 YWLHIV calculated using the Kish Leslie formula [38]. The formula assumed the Z score at 95% confidence interval of 1.96, female-controlled contraceptive prevalence p of 0.5 and precision d of 0.05 to produce a sample size of 385 participants. After 10% adjustment for non-response, the final sample size came to 424 YWLHIV. The initial sample size for the qualitative in-depth interviews will be 26 YWLHIV. Further sampling will be determined by the data saturation which is a point at which further sampling does not yield any new data that was not already mentioned by the previous respondents [39].

### Sampling methods and participant’s recruitment

Stratified random sampling method will be used. The total list of the YWLHIV on ART from the six public health facilities will first be established from the health facilities and then stratified into three strata namely regional referral hospital patients, health center level IV patients and health center level III patients. The three strata of health facilities are considered because they offer varied spectrum of ART, reproductive and contraceptive services and also have differences in the number and professional education level of the health workforce. Proportionate to size number of participants from each stratum will be selected by probability sampling. The participants for the qualitative in-depth interviews (IDIs) will be a sub-sample of the YWLHIV who will be purposively sampled based on the criteria that they are ever users or non-users of the female-controlled dual protection methods. The YWLHIV participants will first be identified or recruited from the patient registers at the six public health facility-based ART clinics where they receive their routine HIV ARV drugs, care and treatment. The identified YWLHIV will be listed into a sampling frame by the aforementioned strata for sampling. The sampled participants will be traced at the ART clinic on their clinic appointment dates or to their respective homes with the help of the ART linkage facilitators, the village health teams (VHTs) and the village political leader also known as the Local Council I (LCI) chairpersons.

### Data collection tools

The data collection tool will be the questionnaire for the quantitative study. The items of the questionnaire are described under the measures of the variables. An IDI guide will be the tool for embedded qualitative study. The IDI contains items for deeper exploration of the women’s experiences and perceptions regarding the female-controlled dual protection methods including its awareness, access, benefits, advantages, disadvantages, barriers and challenges. Female trained Research Assistants (RAs) with nursing and midwifery professional background will collect the data for both the quantitative and the qualitative sub studies.

### Data collection procedures

Data collection was still ongoing at the time of first submission of this study protocol to the journal (on the 14th March 2023). The data collection sessions for both quantitative and qualitative studies will be conducted in private settings within the premises of the ART clinics to ensure privacy. All questionnaires will be administered by interviewer during the face-to-face meetings. The administration of the questionnaire by interviewer will ensure collection of quality data and also prevent risk for missing data. Data collection from in-school YWLHIV will be handled in private and confidential manner during holidays when they visit the ART clinics for their drug refills. Similarly, YWLHIV who miss their ART clinic appointments will be specifically traced to their homes and provided transport facilitation to come for the data collection session at the ART clinic premises. The ART linkage facilitators or the VHT will be the ones to trace the selected participants to their homes and provide them with the necessary transport facilitation to come for the data collection at the ART clinic premises. The transport facilitation will be covered by the research budget at no cost to the participants. Ensuring all questionnaires are administered during physical face to face meetings at the ART clinic in a private and confidential space will mitigate the unofficial involvement of third parties such as the male partners or husbands or other family members or community members in the data collection and breaching privacy and confidentiality of the data. If the YWLHIV on their own accord decides to disclose their research participation to their male partners or husbands, the male partners or husbands will be counselled by the RAs that they will be involved in the next phase of the research that will target the male partners or husbands of the YWLHIV as research participants. The questionnaires will be anonymous and will take about 1 hour to complete. Similarly, the interviews will take about 1 hour. The interviews will be administered in the local language (Langi) by the RAs who will be trained for the tasks. The interviews will be recorded using audio-recording devices.

### Primary outcome for the quantitative part of the study

The primary outcome is the use of the female-controlled dual protection method in the past 12 months.

### Secondary outcomes for the quantitative part of the study

The secondary outcomes are (a) the use of male-dependent dual protection method in the past 12 months, (b) unintended pregnancy, and (c) unmet need for contraception.

### Exposure variables and covariates for the quantitative part of the study

The exposure variables and covariates for the quantitative part of the study are (a) awareness about modern contraceptive methods, (b) proximity to the source or the providers of contraceptive methods, (c) the woman’s desire to bear children, (d), the male partner’s desire to bear children, (e) awareness of HIV positivity of the male partner even before first sexual intercourse, (f) preferences of safe conception methods, (g) perceptions of dual protection method, (h) perceptions of the safe conception methods, and (i) socio-demographic characteristics of the women particularly the age, tribe, religion, marital status, urban or rural nature of the residential address, distance from the nearest health facility, educational level, occupation, and income level.

### Phenomenon of interest for the qualitative part of the study

The phenomenon of interest for the qualitative part of the study are the women’s experiences and perceptions of the dual protection and safer conception methods. For the ever or current users of the dual protection and safer conception methods, experiences and perception phenomenon will entail exploration of the motivating or demotivating factors behind their use, choices, beliefs, and perspectives regarding the benefits, advantages, disadvantages, challenges, and barriers of using the dual protection and safer conception methods for prevention of both HIV transmission and unintended pregnancies. For nonusers, the experiences and perception phenomenon will entail exploration of the reasons for nonuse, beliefs, perspectives, and experiences regarding the benefits, advantages, disadvantages, challenges, and barriers of using the dual protection and safer conception methods for HIV prevention and unintended pregnancies.

### Definitions and measures of the variables

The study aims to measure several variables related to the use of dual protection methods, unintended pregnancy, unmet need for contraception, conception desires, potential predictors, and experiences/perceptions. To measure the use of female-controlled dual protection methods, women will be asked about the methods they use to protect against unintended pregnancy and HIV. They will describe how they use the method, the duration of use, and recent usage. Similarly, women will be asked about the male-dependent dual protection methods they use and provide details on their usage. Women who do not use any dual protection methods will be asked about their reasons for not using them. Regarding unintended pregnancy, women will be asked if they have been pregnant in the past 12 months and whether it was intended or not. They will also indicate if the pregnancy was mistimed, unwanted, or if they had an ambivalent attitude. Additionally, they will report if the pregnancy was unintended for the male partner. Unmet need for contraception will be determined by asking women if they wanted contraceptive methods to prevent unintended pregnancy or to delay/space their children but were unable to obtain them. Women will also be asked about their desire to bear children despite living with HIV and their preferred safe conception methods. Potential predictors for dual protection method use will be explored by asking women who do not use dual protection methods about their reasons for not using them. Participants will also be asked about their awareness and proximity to sources/providers of contraceptive methods, their and their male partner’s desires to bear children, and their perceptions of dual protection and safe conception methods. The women’s sociodemographic characteristics particularly their age, tribe, religion, educational level, marital status, urban or rural nature of the residential address, distance from the nearest health facility, occupation and income level will be collected as well. Experiences and perceptions of dual protection methods will be assessed by asking participants about their beliefs, perspectives, and experiences regarding the benefits, advantages, disadvantages, challenges, and barriers of using such methods for HIV prevention and unintended pregnancies. This includes whether they have ever used or currently use female-controlled dual protection methods, and the motivating factors or obstacles behind their choices. The study has adapted measures from a previous study conducted in Uganda and considers potential predictors based on the health belief model used in HIV prevention and PrEP studies [40].

### Data management

The completed questionnaires and audio-recorders with data will be kept under lock and key or password protected only accessible to the research team in the Principal Investigator’s office. Data including audio recordings will be kept for a period of 5-years as per the Lira University research policy and then destroyed. Data on hard copy data collection tools in this case questionnaire and notebooks will be destroyed by shredding and then burning. Softcopy databases will be destroyed by deleting it from the computers and other external storage device in this case flash discs. Every participant will receive refreshment and compensation for their participation in the study.

Quantitative data will be double entered by two Data Clerks in data entry face in EpiData entry 3.1. software. Missing continuous and categorical data will be imputed using mean replacement and the last value carried forward methods respectively. Validated dataset will be exported from EpiData to the Statistical Package for Social Sciences (SPSS) version 23.0 for statistical analysis. Qualitative data on the experiences and perceptions regarding the use of the dual protection methods will be audio-recorded and transcribed verbatim. Similarly, both the audio-recorders and the transcripts will be kept under lock and key in the Principal Investigator’s office.

### Data analysis

The participant’s demographic characteristics will be summarized into frequency counts, percentages and point estimates using the descriptive statistics. Frequency count and percentages will be calculated for the primary and secondary outcomes. There are two YWLHIV groups of interest. These are (1) those aware or unaware of contraceptive methods for YWLHIV and (2) those aware or unaware of safe conception methods for YWLHIV. Group differences on the outcome will be compared using Chi square (or Fisher’s exact) tests. Correlation or Chi square statistics followed by multiple logistic regression will be used to identify independent predictors for the use of the female-controlled dual protection methods. The level of significance of 0.05 and 95% confidence interval will be considered.

Qualitative data on transcripts will be manually coded by two investigators working in parallel. Code trees will be drawn and reconciled for discrepancies between the two investigators. Code trees will be analysed for themes and categories in accordance with the consolidated guidelines for reporting qualitative study [41]. Findings will be presented in descriptive format with direct quotations from the participants [41].

### Study limitations and mitigation measures

The use of interviewer-administered questionnaire for data collection in this study is prone to social desirability bias more so on sensitive topic in this case sexual health. This will be minimized by the use of female RAs with nursing and midwifery educational background (RAs) to administer the questionnaire in private and confidential spaces within the health facility. Training of the RAs on deep probing and the use of triangulation of data collection methods in this case questionnaire and in-depth interviews are other mitigation measures against potential social desirability bias. There is also the risk of measurement errors as participants will be asked to remember their sexual health practices in the past 12 months. Measurement errors will be minimized through training of the RAs on deep probing and triangulation of data collection methods in this case the use of both questionnaire and in-depth interviews where applicable. The lack of laboratory testing for the presence of hormonal contraceptives and ARV drugs in the blood of the YWLHIV and also the lack of data from the perspectives of the male partners of the YWLHIV are other potential study limitations. The potential threat to internal validity arising from the lack of laboratory testings and the lack of data from the perspectives of the male partners in the study will be minimized through deep probing and triangulation of data collection methods in this case the use of both questionnaire and in-depth interviews.

### Ethical considerations

The ethical approval for the study was obtained from the Gulu University Research Ethics Committee (GUREC) with the approval number GUREC -2022-309. We further conducted research registration and clearance with Uganda National Council of Science and Technology (UNCST). Potential participants will be first screened for eligibility to participate in the study. Informed consent shall be obtained from all the participants aged 18 years and above. Assent will be obtained from participants below 18 years plus informed consent from their parents or guardians. The participants shall be provided with information about the purpose of the study, methods of data collection, study time frame, risks, benefits, and freedom to refuse or withdraw participation from the study without penalties before enrolment into the study and data collection. The RAs will moderate the informed consent process with the participants. Informed consents will be obtained by written signature or thumb print. The participants will be assured of confidentiality of the data. The data collection tool shall be anonymized. Privacy shall be assured by conducting the data interviewer-administered questions in spaces within the hospital free from third party interference. Data on hard copy questionnaire shall be kept under lock and key only accessible to the research team. Electronic database shall be password protected and only accessible to the research team. Furthermore, all methods will be performed in accordance with the Declaration of Helsinki and other relevant guidelines and regulations. Each participant will be provided a compensation of Ugandan shilling 10,000 (equivalent to US$ ~3) for their participation in the research.

## Supporting information

S1 File (PDF)

## List of abbreviations

AGYWLHIV: Adolescent Girls and Young Women Living with HIV
AIDS: Acquired Immune Deficiency Syndrome
AMLHIV: Adolescent Mothers Living with HIV
ART: Anti-Retroviral Therapy
ARV: Anti-Retroviral Drugs
HIV: Human Immune Deficiency Virus
IDI: In-Depth Interviews
LRRH: Lira Regional Referral Hospital
LUH: Lira University Hospital
PEP: Post exposure prophylaxis
PrEP: Pre-Exposure Prophylaxis
SPSS: Statistical Package for Social Sciences
SSA: Sub Saharan Africa
STDs: Sexually Transmitted Diseases
