# Supplementary material for: Female-controlled dual protection methods: Prevalence, predictors, experiences and perceptions among young women living with HIV in northern Uganda–A mixed-method study protocol
Source: PLoS One. 2023 Oct 9;18(10):e0290338. doi: 10.1371/journal.pone.0290338 (PMC10561857; doi:10.1371/journal.pone.0290338)
Supplement: S1 File — (PDF) [file pone.0290338.s001.pdf]

ELIGIBILITY CHECK VERSION 4.0.

Sn	Eligibility Check	Yes	No
A1	Have you ever played sex in your lifetime? NB – tick yes without asking if the participant has a child or is breast feeding.		
A2	Can you conceive a baby? I mean are you fertile? NB – tick yes without asking if the participant has a child or is breast feeding.		
A3	Do you feel you can you use any modern contraceptive methods including hormonal methods without complications?		
A4	Did the health workers ever diagnose you as having mentally illness?		
A5	Is the participant observably healthy to withstand the study procedures?		

QUESTIONNAIRE FOR AGYWLHIV VERSION 4.0.

Questionnaire number …………………………

B	Section B: Participant's Demographic Characteristics	
B1	How old are you in completed yearr?	
B2	What religion are you?	(1). Catholic<br>(2). Anglican<br>(3). Protestant<br>(4). Muslim<br>(5). Others specify……………………………
B3	What ethnic tribe are you?	(1). Langi<br>(2). Acholi<br>(3). Itesot<br>(4). Bagisu<br>(5). Others specify ……………………………
B4.	What is your educational level	(1). No formal education<br>(2). Primary School<br>(3). Secondary school<br>(4). Certificate or Diploma Tertiary School<br>(5). Bachelors<br>(6). Master<br>(7). Others specify ……………………………
B5	What is your most important source of earning/ money/ income during the past 12 months?	(1). Domestic remittances e.g. gifts from parents, siblings, relatives, male partner or husband<br>(2). Subsistence agriculture<br>(3). Commercial farming<br>(4). Wage/ Salary employment<br>(5). Non-agricultural enterprises<br>(6). Others specify………………………………
B6a	How much net income in Ugandan shillings do you earn per month from your	

	main occupation or source of income?	
B6b	How much net income in Ugandan shillings do you earn per month from your main occupation or source of income?	1) < 83,332<br>2) 83,333 – 416,665<br>3) 416,666 – 833,332<br>4) 833,333 – 1,249,999<br>5) 1,250,000 – 1,666,665<br>6) 1,666,666 – 2,083,332<br>7) 2,083,333 and above
B7	B7. What is your current marital status?,	(1). Single/ Never married<br>(2). Married (culturally, in church or legally or cohabiting)<br>(3). Previously married but Separated/ Divorced/ Widowed<br>(4) Others specify<br>…………………………………………
B8a	The year and your age at the first sexual intercourse	…………………..<br>…………………………….
B8b	The year and your age when you started having the sexual affairs with your current main boyfriend, husband or male partner?	……………………<br>……………………………..
OBSTERICAL HISTORY		
B9	How many times have you ever gotten pregnant?	……………………
B10	How many times have you ever given birth including premature birth?	……………………
B11.	How many of your pregnancies resulted into miscarriages and abortions?	……………………
B12.	How many of your children are currently alive?	……………………
B13.	What is current residential address?	District or City name: …..………………<br>Sub county/ Division ………………….
B14.	What is the urbanization status of your residential area?	1). Rural area<br>(2). Suburb or small town<br>(3). Municipality or medium town<br>(4). Large city
FAMILY PLANNING METHODS AVAILABLE		
B15.	How far in kilometers is your current residential address from the nearest health facility	

	…………………………… that provides family planning methods e.g. injections whether it being private for profit, private not for profit or government health facility?<br><br>Note for Research Assistants: confirm with Google Map the distance from the participants village to the nearest health facility that provides family planning methods	
B16.	In your residential or workplace area, are there sources/ places/ providers where the adolescent girls and young women like you or their male partners can obtain modern family planning methods whenever they need it?	0. No …………….<br>1. Yes …………..
B17.	What family planning method sources/ places/ providers exists in your residential or workplace area? Mention all that are available<br>(1). Village health teams (community health workers),<br>(2). Private for profit clinics or medical centers<br>(3)Community-based drug shops or pharmacies<br>(4) Government and or private health facility<br>(5) Others specify	0. No ……. 1. Yes ………<br><br>0. No …….. 1. Yes ………<br>0. No …….. 1. Yes ………<br>0. No …….. 1. Yes ………<br>………………………………………
B18.	What forms of Information, Education, Communication materials and or counseling did you ever receive about family planning methods?	1. Radio program 0. No …… 1. Yes ………<br>2. TV program 0. No ……. 1. Yes ………<br>3. Poster 0. No ……. 1. Yes ………

		4. Billboard 0. No ……. 1. Yes ………<br><br>5. Others specify ……………………………
B19	What modern family planning methods are available and offered to PLHIV from the ART clinic where you usually obtain your ARV drug refill?	
B20.	Which modern family planning methods have you ever received from the ART clinic where you usually obtain your ARV drug refill?	
**ALCOHOL AND DRUG USE**		
B21.	How often do you drink alcohol?	0. Never 1. Occasional 2. Weekly 3. Daily
B22.	Specify the drugs you use	…………………………………………………… ………………..
B23.	How often do you use the drugs?	0. Never 1. Occasional 2. Weekly 3. Daily
B24	How often do you smoke tobacco?	0. Never 1. Occasional 2. Weekly 3. Daily
**HIV ACQUISITION**		
B25.	How did you acquire the HIV infection?	(1). Mother to child transmission<br>(2). Blood transfusion with infected blood<br>(3). Needle stick injuries with infected person's blood<br>(4). Unprotected sexual intercourse with an infected person<br>(5). Others specify ………………………………
B26.	In which year and at what age did you discover you are HIV infected?	…………………… ……………………..
B27.	How did you discover you were HIV infected?	(1). HIV testing at the health facility<br>(2). Blood donation testing<br>(3). HIV testing at community-based health outreach event<br>(4). Self-testing<br>(5). Others, specify……………………………….
**ART**		
B28.	In which year and at what age did you start receiving the ART or ARV drugs?	………………………… ………………………..

B29.	How many types of medicines do you usually receive from the ART clinic per visit?	
**SEXUAL ACTIVITY**		
B30.	About how often did you have sex during the past 12 months?	(0). Not at all<br>(1). Once or twice<br>(2). Once a month<br>(3). 2-3 times a month<br>(4). Weekly<br>(5). 2-3 per week<br>(6). 4 or more per week<br>(7). Unsure of the frequency
B31.	How long ago (in days or weeks or months) was your last/ most recent sexual intercourse	
**SECTION C: PRIMARY OUTCOME (DUAL PROTECTION)**		
C1a.	What methods under your control as a woman living with HIV do you use to prevent both unintended pregnancy to yourself and HIV transmission to your male partner during sex?	0. None (**Skip to C7**)<br>1. Hormonal contraceptive ……………………… (method name) + Daily oral ARV drugs (PrEP) both by me female partner LHIV<br>2. Others specify ………………………………….. ……………………………………………… …………..
C1b.	**If using dual protection method, describe how exactly you use** the aforementioned woman controlled dual protection methods	
C2.	If using dual protection methods, for how long have you used the woman – controlled dual protection method?	Year started Years/ months used<br><br>…………………. ……………………………..
C3.	Did you use the woman – controlled dual protection method at your last/ most recent sex?	
C4.	Did you use the woman – controlled dual protection method in the past 3 months?	

C5.	Did you use the woman – controlled dual protection method in the past 6 months?	
C6	Did you use the woman – controlled dual protection method in the past 12 months?	
C7	**If not using any of the women – controlled dual protection** methods to prevent both unintended pregnancy and HIV transmission during sexual intercourse, why not using?	
**Section D: Secondary Outcomes**		
D1a.	What methods that are dependent on your male partner do you use to prevent both unintended pregnancy to yourself and HIV transmission to your male partner during sex?	0. None (Skip to D7)<br>1. Condom only by my male partner<br>2. Condom + Daily oral ARV drugs (PrEP) by my male partner<br>3. Condom + 28 days' course of oral ARV drugs (PEP) by my male partner<br>4. Hormonal contraceptive …………………… (method name) by me female partner LHIV + 28 days' course of oral ARV drugs (PEP) by my male partner<br>5. Hormonal contraceptive …………………… (method name) by me female partner LHIV + Daily oral ARV drugs (PrEP) by my male partner<br>6. Hormonal contraceptive …………………… (method name) by me female partner LHIV + Daily oral ARV drugs (PrEP) by me female partner LHIV + Daily oral ARV drugs (PrEP) by my male partner<br>7. Hormonal contraceptive …………………… (method name) by me female partner LHIV + Daily oral ARV drugs (PrEP) by me female partner LHIV + 28 days' course of oral ARV drugs (PEP) by my male partner<br>8. Hormonal contraceptive …………………… (method name) by me female partner LHIV + Daily oral ARV drugs (PrEP) by me female partner LHIV + condom by male partner

		9. Others specify …………………………………..<br>………………………………………………………………………
D1b.	**If using any of the men-dependent dual protection methods,** describe how exactly you use it	
D2.	For how long have you used the woman – controlled dual protection method?	Year started Years/ months used<br>……………… ………………
D3.	Did you use the male partner – controlled dual protection method at your last/ most recent sex?	
D4.	Did you use the male partner – controlled dual protection method in the past 3 months?	
D5.	Did you use the male partner – controlled dual protection method in the past 6 months?	
D6	Did you use the male partner – controlled dual protection method in the past 12 months?	
D7	**If not using any of the men – dependent dual protection method** (e.g. condom, Daily ARVs by male partner even on days preceding the sexual intercourse for pregnancy, 28 days course of ARV after the sexual intercourse for pregnancy), why not using?	
**E**	**Unmet Needs for Contraception**	
E1.	Have you ever wanted a contraceptive method to avoid or prevent unintended pregnancy but failed to get it?	0. No 1. Yes
E2.	Have you ever wanted a contraceptive method to delay or space your children but failed to get it?	0. No 1. Yes

E3	If yes, what made to fail to get the contraceptive method when you wanted it to avoid or prevent unintended pregnancy or delay or space your children?	
**F**	**Unintended Pregnancy**	
F1.	How long ago was your last/ most recent sex without being on family planning methods including condom?	
F2.	Did you get pregnant in the past 1 year?	0. No 1. Yes
F3.	If yes, did you intend to have the pregnancy at that time?	0. No, unintended 1. Yes, intended
F4.	If unintended, was the unintended pregnancy just	(1). Mistimed (2). Unwanted (3). You didn't care
F5.	If unintended, was it unintended pregnancy to your male partner?	0). No (1). Yes (2). Don't know
F6.	What was the unintended pregnancy outcome?	(0). Miscarriage or aborted (1). Still pregnant (2). Premature birth (3) normal live birth (4) still birth (5) others specify ………………………………
**G**	**HIV Transmission to male partner**	
G1	How long ago did you start dating with your current main male sexual partner?	
G2	How long ago did you actually start having sex with your current main male sexual partner?	
G3	What was your HIV status before meeting with your current main male sexual partner?	(0). Negative (1). Positive (2) Unknown
G4	Was your HIV status disclosed to your current main male sexual partner before the first sexual intercourse with him	(0). No (1). Yes
G5	What was the HIV status of your current main male sexual partner before meeting you?	(0). Negative (1). Positive (2) Unknown

G6	Was the HIV status of your current main male sexual partner disclosed to you before first sexual intercourse with him?	(0). No (1). Yes
G7	What is the current HIV status of your main male sexual partner after having several months of sexual relationship with you?	(0). Negative (1). Positive (2) Unknown
H	**Section H: Potential Factors and Predictors for the Use of the Dual Protection Methods**	
H1	Do you get sexual feelings	0. No 1. Yes
H2	Do you desire to bear children?	0. No 1. Yes
H3	If yes, what is your desired number of children to bear?	
H4a	If yes, explain why you desire to bear children even though you are AGYWLHIV	
H4b	If no, explain why you don't desire to bear children even though you are AGYWLHIV	
H5	Does your male partner desire to father children?	0. No 1. Yes 2. Don't know
H6	What is the desired number of children of your current male partner desire?	
H7	What dual protection methods for women living HIV do you know of	0. None<br>1. Hormonal contraceptive ……………………… (method name) + Daily oral ARV drugs (PrEP) both by me female partner LHIV<br>2. Others specify …………………………………..
H8	What are your views on the benefits or advantages of using the dual protection methods (hormonal contraceptive + Daily oral ARV drugs) for women living with HIV?	
H9	What are your views of the disadvantages and challenges of using the dual protection methods (hormonal contraceptive + Daily oral	

	ARV drugs) for women living with HIV?	
H10	What safer conception methods for women living with HIV do you know of?<br><br>Note for Research Assistants: Safer conception methods for women living with HIV are methods that can allow the women to get pregnant without transmitting HIV to their male partners in the process of conception.	0. I don't know<br>1. Daily oral ARVs by female partner LHIV before the sexual intercourse meant to get pregnant<br>2. Daily oral ARVs by male partner before the sexual intercourse meant to get impregnate<br>3. 28 days' course of ARVs by the male partner after the sexual intercourse meant to impregnate<br>4. Artificial insemination<br>5. Invitro fertilization<br>6. Others specify ………………………………
H11	What are your views on the benefits or advantages of using the safer conception methods (e.g. Daily oral ARVs by the male partner, 28 days' course of ARVs by the male partner or artificial insemination) for women living with HIV?	
H12	What are your views on the disadvantages and challenges of using the safer conception methods (e.g. Daily oral ARVs by the male partner, 28 days' course of ARVs by the male partner or artificial insemination) for women living with HIV?	

H13. Any other comment or views about dual protection against unintended pregnancy and HIV transmission that you would like to share?
……………………………………………………………………………………………………
……………………………………………………………………………………………………
THE END, THANK YOU FOR PARTICIPATING IN THE RESEARCH

IN-DEPTH INTERVIEW GUIDE FOR AGYWLHIV VERSION 4.0.

Unique ID number: …………………………………………………………….

Participant's category: …………………………………………………………

Participant's Demographic Data

1. ART clinic name: …………………………………………………………
2. Age: …………………………………………………………
3. Year and your age at HIV diagnosis: …………………………………………………………
4. Year and your age at initiation of ART: …………………………………………………………
5. Year and your age at first sexual intercourse: …………………………………………….
6. Year and your age at first sexual intercourse with the current sexual partner: …………
7. Marital status: …………………………………………………………
8. Address: ………………………………………………………………………….
9. Education level: ………………………………………………………………….
10. Occupation: ………………………………………………………………………..
11. Religion: …………………………………………………………………………..
12. Your monthly income: ……………………………………………

Dual Protection Benefits and Advantages
Female – controlled dual protection method

C5a. What women-controlled dual protection methods that prevents both unintended pregnancy and HIV transmission to male partners do you know of?

C5b. Have you recently (within the past 12 months) used the dual protection method to prevent both unintended pregnancy and HIV transmission to your male partner? …………………..

C5c. If yes you are using the women-controlled dual protection methods, tell me how exactly you use the women-controlled dual protection methods that you use to prevent both unintended pregnancy and HIV transmission to male partners? ………

> **Probes:** describe how exactly you use it, when exactly did you start using it, how did you get to know of it (source of knowledge), from where did you obtain the methods (method source), how often do you use it, when do you not use it and why, what roles do your male partner play during the use of the dual protection methods, what exactly prompted you to start using it?

C5d. If no you are not using the woman-controlled dual protection method, tell why not? …..

Probes: Explain how exactly the challenge or barrier you have given is blocking you or making it not possible for you to use the dual protection methods? How did the challenge or barrier arise? Is the challenge also affecting other AGYWLHIV?

C5e. Are there solutions or interventions being implemented to address the challenge or barriers you are facing regarding use of women-controlled dual protection method?

Probes: What solutions or interventions exactly, whom and when did it start, who are the main target beneficiaries, have you benefited from it, has it helped you?

C6. What are your views on the benefits or advantages of using the woman-controlled dual protection methods that prevents both unintended pregnancy and HIV transmission to male partner? ……………………………………………………………………………………

C7. What are your views on the disadvantages and challenges of using the woman -controlled dual protection methods that prevents both unintended pregnancy and HIV transmission to male partners? ……………………………………………………………………………………

C8. What challenges and barriers did you personally encounter in your attempt to use the woman – controlled dual protection methods to prevent both unintended pregnancy and HIV transmission to your male partner? ………………………………………………….

C9. Do you have any other comment or views about the dual protection that prevents both unintended pregnancy and HIV transmission that you would like to share? **………………………**

THE END, THANK YOU FOR PARTICIPATING
